# Differential Expressions of the Alternatively Spliced Variant mRNAs of the µ Opioid Receptor Gene, *OPRM1*, in Brain Regions of Four Inbred Mouse Strains

**DOI:** 10.1371/journal.pone.0111267

**Published:** 2014-10-24

**Authors:** Jin Xu, Zhigang Lu, Mingming Xu, Grace C. Rossi, Benjamin Kest, Amanda R. Waxman, Gavril W. Pasternak, Ying-Xian Pan

**Affiliations:** 1 Department of Neurology and Molecular Pharmacology and Chemistry Program, Memorial Sloan Kettering Cancer Center, New York, New York, United States of America; 2 Department of Psychology, Long Island University, Post Campus, Brookville, New York, United States of America; 3 Department of Psychology and Center for Developmental Neuroscience, City University of New York, Staten Island, New York, United States of America; National University of Singapore, Singapore

## Abstract

The µ opioid receptor gene, *OPRM1*, undergoes extensive alternative pre-mRNA splicing in rodents and humans, with dozens of alternatively spliced variants of the *OPRM1* gene. The present studies establish a SYBR green quantitative PCR (qPCR) assay to more accurately quantify mouse *OPRM1* splice variant mRNAs. Using these qPCR assays, we examined the expression of *OPRM1* splice variant mRNAs in selected brain regions of four inbred mouse strains displaying differences in µ opioid-induced tolerance and physical dependence: C56BL/6J, 129P3/J, SJL/J and SWR/J. The complete mRNA expression profiles of the *OPRM1* splice variants reveal marked differences of the variant mRNA expression among the brain regions in each mouse strain, suggesting region-specific alternative splicing of the *OPRM1* gene. The expression of many variants was also strain-specific, implying a genetic influence on *OPRM1* alternative splicing. The expression levels of a number of the variant mRNAs in certain brain regions appear to correlate with strain sensitivities to morphine analgesia, tolerance and physical dependence in four mouse strains.

## Introduction

**Figure 1:**
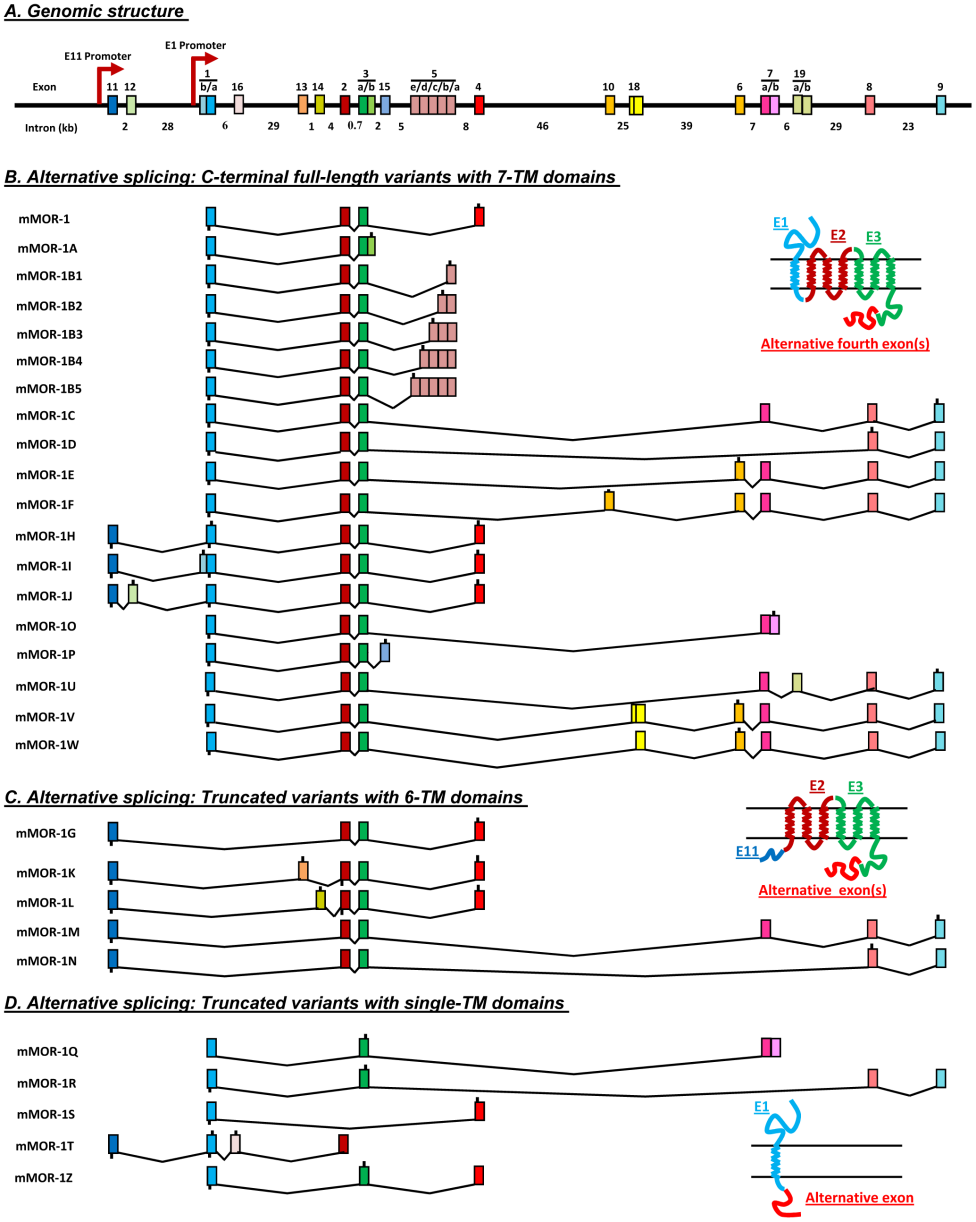
Schematic of the mouse *OPRM1* gene structure, alternative splicing and predicted protein structures. A, Genomic structure of the mouse *OPRM1*gene. Exons and introns are showed by boxes and horizontal lines, respectively. Intron size is indicated below the introns as kilobases (kb). Promoters are showed by arrows. Exons are numbered based upon the published data. B, Alternative splicing of C-terminal splice variants with 7-TM domains. Translational start and termination sites are indicated by downward and upward lines on exon boxes, respectively. The lines between exons are introns that are spliced out during splicing. Predicted receptor structures are inserted at the right with colored bars indicating different coding exons. C, Alternative splicing of truncated variants with 6-TM domains. D, Alternative splicing of truncated variants with 1-TM domain.

Morphine and most clinical analgesic agents, as well as heroin, act primarily through µ opioid receptors [1]. However, their actions, such as analgesia, tolerance, physical dependence and other side-effects vary among patients [2]–[4]. Similar effects have been observed in animal models [5]–[9]. These observations are consistent with the concept of subtypes of µ opioid receptors that was initially proposed by pharmacological studies [10]–[12] and subsequently confirmed with the isolation of a multitude of µ opioid receptor variants generated by alternative splicing [13]–[17] in rodents and humans despite the presence of a single µ opioid receptor gene, as determined by chromosomal mapping [18]–[21] and genomic sequencing. Alternative pre-mRNA splicing is a major mechanism for generating protein diversity and is extensively used by the µ opioid receptor gene. To date, studies show that the mouse, rat and human *OPRM1* genes generate 29, 16 and 19 splice variants, respectively, with similar patterns (Fig. 1) (see reviews: [16], [22] ). These splice variants can be categorized into three major groups based upon receptor structure: 1) Full-length carboxyl (C-) terminal variants with 7-transmembrane (7-TM) domains; 2) Truncated variants containing 6-TM domains; and 3) Truncated variants containing a single TM. The full length 7-TM C-terminal variants differ in their region-specific expressions, receptor phosphorylation, internalization, and post-endocytic sorting [23]–[31]. Furthermore, the intrinsic activities of µ drugs vary from variant to variant [32]. The significance of the exon 11-associated 6-TM variants is illustrated by their role in the analgesic actions of opioids such as morphine-6β-glucuronide (M6G), fentanyl and heroin [33], as a novel new class of opioid analgesics lacking many of the side-effects of traditional opiates [34].

The sensitivity of mice towards µ opioids on a variety of phenotypic measures is genotype dependent [35]–[40]. For example, surveys of inbred mouse strains reveal differences in morphine dependence, assessed with naloxone-precipitated withdrawal jumping scores, and tolerance, as seen by analgesic potency shifts after chronic morphine administration [35], [36], [41]. 129P3/J mice are highly refractory to morphine tolerance and naloxone-precipitated withdrawal, whereas C57BL/6J and SWR/J mice develop significant morphine tolerance and physical dependence. These inbred mouse strains also show similar differences in naloxone-precipitated withdrawal jumping scores toward other µ opioids including heroin [37], M6G and methadone [42]. However, it is not clear how the genetic background of these strains influences the expression of the *OPRM1* gene, a primary candidate responsible for the actions of µ drugs seen in these strains.

Our previous studies suggested region-specific alternative splicing of the *OPRM1* gene among brain regions using regular RT-PCR approaches [25]–[28]. However, regular PCR can only provide estimates of quantification due to technical limitations. To establish more accurate, reliable and sensitive assays for quantifying *OPRM1* variant mRNAs we have established SYBR green quantitative PCR (qPCR) assays for the *OPRM1* splice variants. Using these qPCR assays, we examined the expression levels of *OPRM1* splice variant mRNAs in selected brain regions of four inbred mouse strains: C56BL/6J (B6), 129P3/J (129), SJL/J (SJL) and SWR/J (SWR).

## Materials and Methods

### Animal

C56BL/6J (B6, stock #: 000664), 129P3/J (129, stock#: 000690), SJL/J (SJL, stock #: 000686) and SWR/J (SWR, stock #: 000689) male mice at 7–8 weeks of age were obtained from Jackson Laboratory. All mice were housed in groups of five, maintained on a 12-h light/dark cycle and given ad libitum access to food and water. All animal studies were approved by the Institutional Animal Care and Use Committee of the Memorial Sloan-Kettering Cancer Center.

### Tissue dissection, RNA extraction and reverse-transcription (RT)

Brain regions including the prefrontal cortex (Pfc), striatum (Str), thalamus (Tha), hypothalamus (Hyp), hippocampus (Hip), brain stem (BS), periaqueductal gray (PAG), and cerebellum (Cb) were dissected on a mouse Plexiglas brain mold using the atlas of Paxinos and Franklin [43] as a reference. The spinal cord (Spc) from L1 to L5 and whole brain were also dissected. The dissected tissues were immediately homogenized in QiAzol Reagent (Qiagen). The homogenates were stored at −80°C until further RNA isolation. 2–3 mice were pooled for each region, and a total of 8–10 mice used for each strain. Total RNAs were extracted using miRNeasy kit (Qiagen) following manufacture protocol. RNA concentrations were determined using a Qubit 2.0 Fluorometer (Invitrogen). RNAs were first treated with Turbo-DNA free reagent (Ambion) following the manufacture’s protocol to remove potentially contaminating genomic DNA, and reverse transcribed with Superscript II reverse transcriptase (Invitrogen) and random hexmers. The first-strand cDNA was then used as templates in SYBR qPCRs.

### Primer design and SYBR green qPCR

Since many exons are shared by different variants, it is difficult to design the short amplicon required for an efficient qPCR using the TaqMan assay for most of the splice variants. SYBR Green qPCR offers more flexibility in terms of amplicon size and thermo-cycle conditions. Using primer design tools in GeneRunner and VectorNTI, we designed appropriate primers for each splice variants, optimized qPCR parameters, such as annealing temperature and extension time, and obtained reasonable amplification efficiency by generating standard curves using 5–7 serial 10-fold dilutions of corresponding cDNAs as templates (Table S1). In most cases, we designed primers targeting exon-exon junctions or crossing at least two exons to obtain specific amplification. Specificities of the primers were verified by qPCRs using plasmid constructs containing corresponding variant cDNAs, and by melting curve analysis and agarose gel analysis after qPCR. Table S1 lists the names, sequences and location of primers, as well as amplicon size, efficiency and PCR parameters, for all 29 splice variants. Two workstations in two separate rooms were used exclusively for qPCR to avoid cross contamination. Three reference genes, succinate dehydrogenase subunit A (SDHA), TATA box binding protein (TBP) and glycerakdehyde 3-phosphate dehydrogenase (G3PDH), were selected to obtain normalization factors using the formula[44]–[46]: Normalization factor (NF) = (C(t)_SDHA_ × C(t)_TBP_ × C(t)_G3PDH_)^1/3^. All qPCRs were performed using HotStart-IT SYBR Green qPCR Master Mix (USB, Affymetrix, INC) with Opticon 2 DNA Engine System (Bio-Rad). Normalized expression (NE) or expression level for each variant was calculated using the formula [44]: NE_variant_ = E^−(C(t)variant – C(t)NF)^ (E^−ΔC(t)^), where E is efficiency. Fold change (FC) was calculated using the formula [44]: FC = NE_variant_/NE_reference_, where a reference was mE1/2 and is was defined as 100%. mE1/2 amplified with primers from exon 1 to exon 2 represented all C-terminal variants, including the original mMOR-1. Although the original mMOR-1 transcribed through the exon 1 promoter is by far the most abundant, it is difficult to design a pair of specific primers for amplifying the exon 1 promoter-driven mMOR-1, since exons 1–4 are shared by several other variants as well.

### Statistical analysis

Data are represented as the mean ± S.E.M. from at least three independent experiments. Two-way ANOVA with Tukey’s multiple comparisons test was used for analyzing qPCR data. Statistical significance was set at *p*<0.05. The results are listed in Table S3.

## Results

### Expression levels of *OPRM1* splice variant mRNAs in four inbred mouse strains

**Figure 2:**
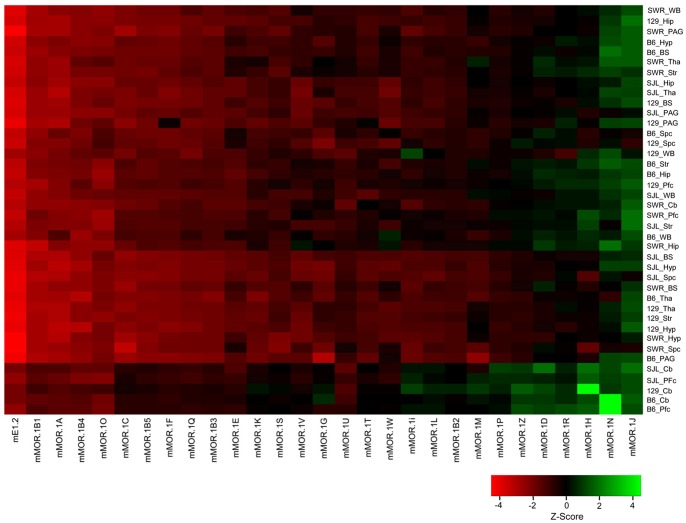
Heatmap of expression levels of *OPRM1* splice variant mRNAs. The heatmap was generated by clustering Z-scores calculated from the values (Log_2_ (E^−ΔC(t)^) of individual variants across different strains/regions using R statistical language (2.15.0) (www.r-project.org), and plotting based upon expression levels among the variants with enhanced heatmap function heatmap.2 from gplots package. Pfc: prefrontal cortex; Str: striatum; Tha: thalamus; Hyp: hypothalamus; Hip: hippocampus; PAG: periaqueductal gray; BS: brainstem; Cb: cerebellum; Spc: spinal cord; WB: whole brain. 129∶129P3/J; B6: C56BL/6J; SJL: SJL/J; SWR: SWR/J. Expression level was indicated by Z-score.

Using the established RT-SYBR green qPCR assays, we examined the expression levels of *OPRM1* splice variant mRNAs in nine brain regions of four inbred mouse strains. The overall expression levels among the variants are shown in a heatmap clustered based upon Z-scores from the values (Log_2_ (E^−ΔC(t)^) of individual variants across different strains/regions, plotting from the highest level (left, red) to the lowest level (right, green) (Fig. 2) (All E^−ΔC(t)^ values are listed in Table S2). The heatmap reveals a wide range of expression levels among the variants, as indicated by Z-score values from<−4 to >4. mE1-2, which represents the entire repertoire of full-length 7-TM variants, was expressed at the highest level in all brain regions and strains. The expression of the other splice variants differed dramatically among the regions and strains.

**Figure 3:**
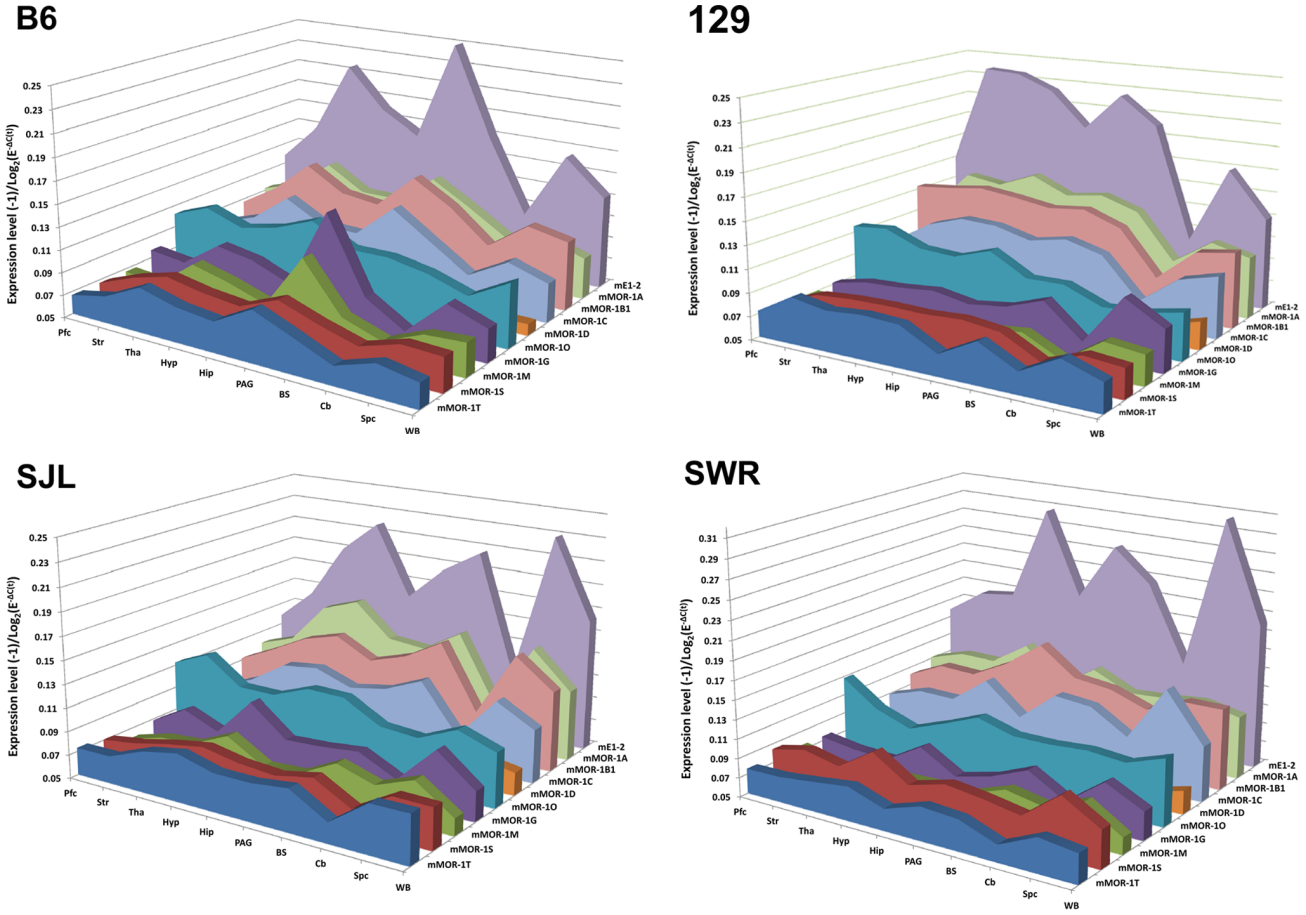
Expression levels of selected ten splice variants in brain regions of four mouse strains. Expression levels of (−1)/(Log_2_ (E^−ΔC(t)^)) of selected variants is plotted across regions using MS excel. Pfc: prefrontal cortex; Str: striatum; Tha: thalamus; Hyp: hypothalamus; Hip: hippocampus; PAG: periaqueductal gray; BS: brainstem; Cb: cerebellum; Spc: spinal cord; WB: whole brain. 129∶129P3/J; B6: C56BL/6J; SJL: SJL/J; SWR: SWR/J.

To visualize expression patterns more closely, we compared five C-terminal splice variants and two 6-TM and two 1-TM variants, as well as mE1-2, by plotting their (−1)/log_2_(E^−ΔC(t)^) values in the brain regions (Fig. 3). The various splice variant expression levels among the brain regions ranged widely. For example, in the hypothalamus of 129 mice, the relative expression levels ranged from 0.1% for mMOR-1M to 22% for mMOR-1A, when their E^−ΔC(t)^ values were used to compare with those of mE1-2 designated as 100% (Fig. 3 & Table S2). The full length C-terminal splice variants mMOR-1A and mMOR-1B1 in 129 mice were highly expressed among most brain regions, corresponding to approximately 10–20% of mE1-2 levels in their E^−ΔC(t)^ values (Fig. 3 & Table S2). On the other hand, in 129 mice the levels of the truncated 6-TM variant mMOR-1G and mMOR-1M and single TM variants, mMOR-1S and mMOR-1T, and the full length mMOR-1D, ranged from about 0.1% to 5% of mE1-2 levels (Fig. 3 & Table S2). Similar expression patterns were observed in other mouse strains (Fig. 3 & Table S2).

### Region-specific expressions of *OPRM1* splice variant mRNAs in four inbred mouse strains

**Figure 4:**
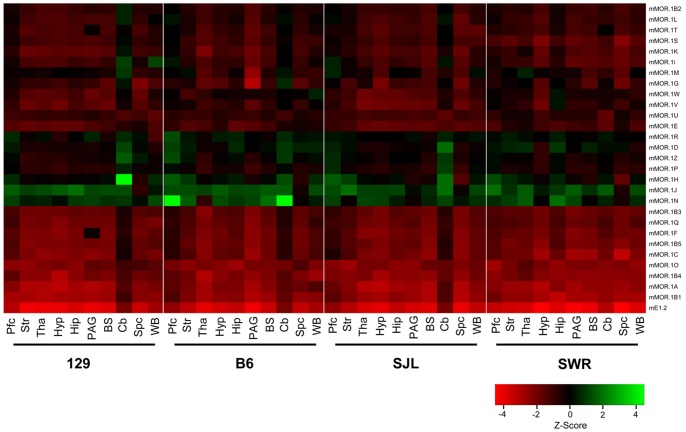
Heatmap of regional expression *OPRM1* splice variant mRNAs. The heatmap was generated by clustering Z-scores calculated from the values (Log_2_ (E^−ΔC(t)^) of individual variants across different strains/regions using R statistical language (2.15.0) (www.r-project.org), and plotting based upon individual brain regions within each mouse strain with enhanced heatmap function heatmap.2 from gplots package. Pfc: prefrontal cortex; Str: striatum; Tha: thalamus; Hyp: hypothalamus; Hip: hippocampus; PAG: periaqueductal gray; BS: brainstem; Cb: cerebellum; Spc: spinal cord; WB: whole brain. 129∶129P3/J; B6: C56BL/6J; SJL: SJL/J; SWR: SWR/J. Expression level was indicated by Z-score.

**Figure 5:**
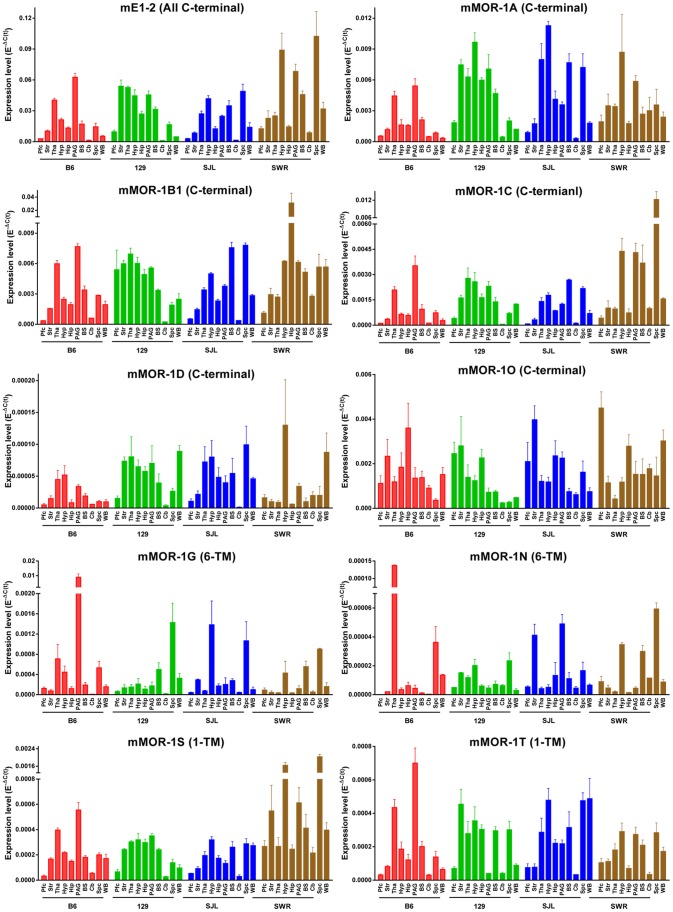
Region-specific expressions of ten-selected *OPRM1* splice variants. Each panel represents the regional expression of one variant in four inbred mouse strains. Red bar: B6 mice; Green bar: 129 mice; Blue bar: SJL mice; Brown bar: SWR mice. Bars represent the mean of E^−ΔC(t)^ values ± S.E.M. Pfc: prefrontal cortex; Str: striatum; Tha: thalamus; Hyp: hypothalamus; Hip: hippocampus; PAG: periaqueductal gray; BS: brainstem; Cb: cerebellum; Spc: spinal cord; WB: whole brain. mE1-2 represents all full-length 7-TM variants. Significant difference was calculated by Two-way ANOVA with Tukey’s multiple comparisons test for each variant (Prism 5.0). The results of the statistical analysis were listed in Table S3. The expression profiles of the other 19 *OPRM1* splice variants are shown in Figures S1 & S2.

Overall, expression levels in whole brain were not predictive of strain differences in specific regions. Within each strain, there are major differences in the regional expression of the variants. The heatmap segregating the different strains shows the overall differential expression pattern of all variants across the brain regions (Fig. 4). Plotting the expression level (E^−ΔC(t)^) for each variant among the regions illustrates the differences quantitatively and provides a visual pattern of regional expression (Figs. 5, S1 & S2). For example, the expression pattern of the full length variant mMOR-1A differed among the four strains. Its levels were high in the thalamus and PAG of B6 mice, with lower levels in the hippocampus and hypothalamus. In contrast, mMOR-1A expression in 129 mice was highest in the hypothalamus with similar high levels in the striatum, thalamus, hippocampus and PAG. In SWR mice mMOR-1A levels were highest in the hypothalamus with lowest levels in the hippocampus.

Within a strain, the regional expression patterns of some of the variants were similar. For example, mMOR-1A, mMOR-1B1, mMOR-1C, mMOR-1G, mMOR-1S and mMOR-1T expression in the B6 mice was highest in the PAG and thalamus (Fig. 5). A similar expression pattern in B6 mice was observed in other eleven variants including mMOR-1B2, mMOR-1B3, mMOR-1B5, mMOR-1F, mMOR-1i, mMOR-1K, mMOR-1L, mMOR-1M, mMOR-1P, mMOR-1Q and mMOR-1Z (Figs. S1 & S2). However, other strains showed different patterns of variant expression among the regions. For example, in SJL mice, mMOR-1*O* expression was highest in the striatum with low levels in the brainstem while mMOR-1C expression levels in these same regions were opposite (Fig. 5).

In general, variant expression was low in the cerebellum and prefrontal cortex, consistent prior *in situ* hybridization studies with the full-length MOR-1 probe [47]. However, this was not uniformly the case. In SWR mice, mMOR-1U expression was greatest in the cerebellum (Fig. S2) while mMOR-1*O* was highest in the prefrontal cortex (Fig. 5).

### Strain-specific expression of *OPRM1* splices variant mRNAs in selected brain regions

**Figure 6:**
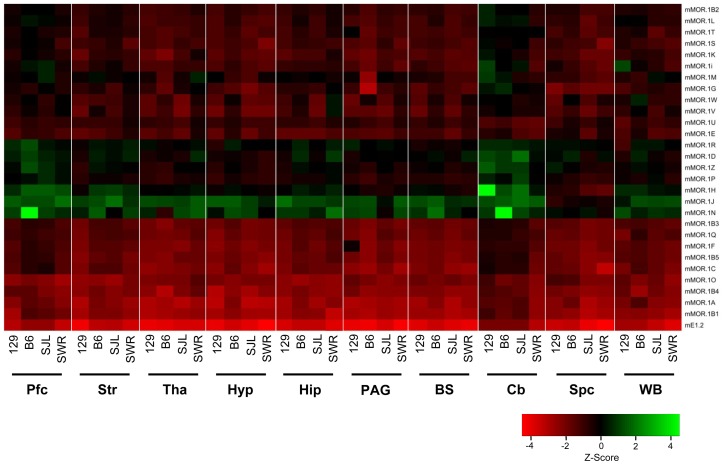
Heatmap of expression of *OPRM1* splice variant mRNAs among four inbred mouse strains. The heatmap was generated by clustering Z-scores calculated from the values (Log_2_ (E^−ΔC(t)^) of individual variants across different strains/regions using R statistical language (2.15.0) (www.r-project.org), and plotting based upon individual strains within each brain region with enhanced heatmap function heatmap.2 from gplots package. Pfc: prefrontal cortex; Str: striatum; Tha: thalamus; Hyp: hypothalamus; Hip: hippocampus; PAG: periaqueductal gray; BS: brainstem; Cb: cerebellum; Spc: spinal cord; WB: whole brain. 129∶129P3/J; B6: C56BL/6J; SJL: SJL/J; SWR: SWR/J. Expression level was indicated by Z-score.

**Figure 7:**
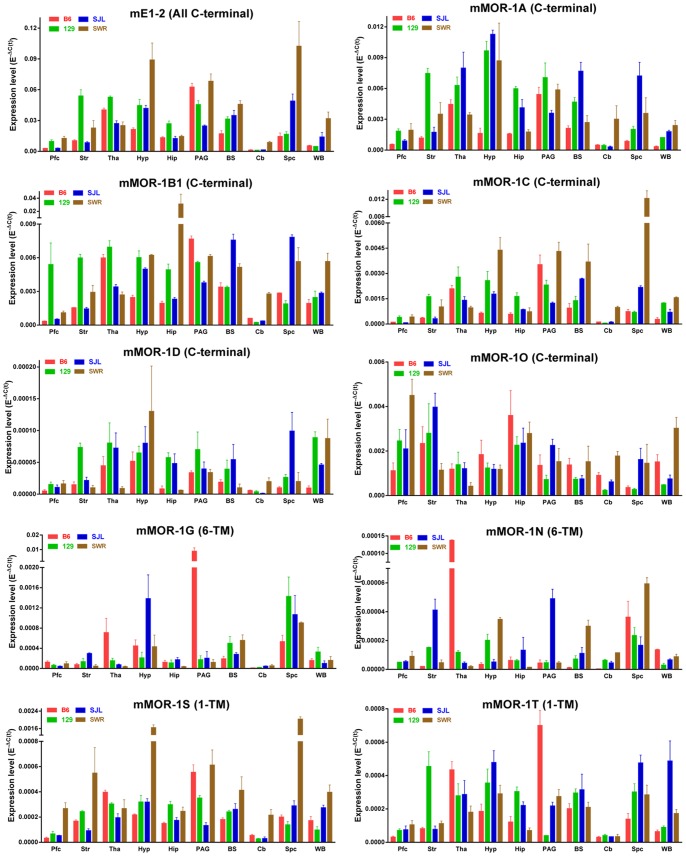
Strain-specific expressions of *OPRM1* splice variants. Each panel represents the expression of one variant in 10 regions of 4 inbred mouse strains. Red bar: B6 mice; Green bar: 129 mice; Blue bar: SJL mice; Brown bar: SWR mice. Bars represent the mean of E^−ΔC(t)^ values ± S.E.M. Pfc: prefrontal cortex; Str: striatum; Tha: thalamus; Hyp: hypothalamus; Hip: hippocampus; PAG: periaqueductal gray; BS: brainstem; Cb: cerebellum; Spc: spinal cord; WB: whole brain. Significant difference was calculated by Two-way ANOVA with Tukey’s multiple comparisons test for each variant. The results of the statistical analysis were listed in Table S3. The expression profiles of the other 19 *OPRM1* splice variants are shown in Figures S3 & S4.

Segregating variant expression by individual regions provides another approach to visualize strain differences in different splice variants, and reveals interesting comparisons (Figs. 6, 7, S3 & S4). The heatmap shows broad differences in expression of individual variants among the strains in most brain regions (Fig. 6), as does quantitative graphing where the expression level (E^−ΔC(t)^) of each variant was plotted by strains within each region (Figs. 7, S3 & S4). Within a region on the heatmap, each column provides the relative expression of the different variants within the strain whereas the horizontal comparisons reveal interstrain differences for a specific variant.

Overall, the expression level of the variants varied among the mouse strains. For example, in the spinal cord, the expression of mMOR-1A in SJL mice was higher than the other strains (Fig. 7). However, in the striatum, 129 mice had the highest expression level. In the thalamus the 6-TM variant mMOR-1N expression was much higher in B6 mice than in other tree mouse strains, contrasting to its expression in the PAG where SJL levels were greater than other three strains (Fig. 7). The expression of the single TM variant mMOR-1S was higher in SWR mice in most brain regions including the spinal cord, hypothalamus, prefrontal cortex, striatum, PAG and brainstem. Although this single TM variant does not directly bind opioid receptors, it is pharmacologically relevant in that it stabilizes the full length mMOR-1 variant and significantly contributes to morphine analgesia [48].

Finally, the expression patterns of the variants within a brain region varied among the strains. For example, the spinal expression of mMOR-1C, mMOR-1i and mMOR-1S in SWR mice was far higher than other strains, while in SJL mice the expression of mMOR-1A, mMOR-1E and mMOR-1L predominated spinally (Figs. 7, S3 & S4). A similar scenario was observed in the PAG where the expressions of mMOR-1B2, mMOR-1G, mMOR-1M, mMOR-1P and mMOR-1T were much greater in B6 mice than the other strains, whereas mMOR-1N had its highest expression in SJL mice (Figs. 7 & S4). In the striatum, mMOR-1A, mMOR-1B2, mMOR-1B3, mMOR-1B5, mMOR-1D, mMOR-1F, mMOR-1Q, mMOR-1T, mMOR-1V and mMOR-1Z were most highly expressed in 129 mice (Figs. 7, S3 & S4). However, mMOR-1N and mMOR-1S were predominately expressed in the striatum of SJL and SWR mice, respectively (Fig. 7). In the thalamus, mMOR-1B3, mMOR-1B4, mMOR-1K, mMOR-1N and mMOR-1R were most highly expressed in B6 mice, whereas the thalamus expression of mMOR-1V and mMOR-1W in SJL mice was much higher than other strains (Figs. 7, S3 & S4). Together, these results suggested strain-specific alternative splicing of the *OPRM1* gene.

## Discussion

The identification of a vast array of splice variants of the µ opioid receptor gene has shown a complexity in opioid pharmacology far beyond what had previously been imagined. Autoradiography provided the earliest means of examining the anatomical distribution of µ binding sites [49]–[52]. The distribution of µ opioid receptors was then examined immunohistochemically and with in situ hybridation following the cloning of the receptor [53]–[63]. While these studies have provided valuable insights into opioid action, they all have limitations. Autoradiographic approaches are functional in the sense that they require active binding of ligand. Since the early studies utilized traditional µ radioligands, they detected all the full length 7-TM variants, but none of the truncated ones, and were not able to distinguish among the 7-TM variants. The early immunohistochemical studies targeted the C-terminus of MOR-1, with most using an epitope encoded by exon 4. These probes will detect the full length variants MOR-1, MOR-1H, MOR-1i and MOR-1J, as well as the 6-TM variant MOR-1G which all share the exon 4 epitope, but none of the other variants. Some studies have examined epitopes from alternatively spliced C-terminals, but they, too, may have labeled more than one species. For example, early studies examined the labeling of a 7/8/9 epitope, assuming it corresponded to mMOR-1C. Yet, subsequent cloning studies identified mMOR-1M, a truncated 6-TM variant that also contains the 7/8/9 epitope. The *in situ* hybridization studies using probes targeting exons 2 and 3 detected all the 7-TM and 6-TM splice variants, but not the single TM ones. Thus, each approach gives a unique set of labeling. Theoretically, qPCR offers the ability to identify an individual mRNA, but even PCR faces selectivity challenges due to both 3′ and 5′ splicing and extensive overlap in sequence among the variants.

We previously used traditional RT-PCR approaches to examine the expression of *OPRM1* splice variant mRNAs in brain regions [25]–[28]. However, these early approaches provided only an approximation of levels since they were not quantitative. Many of these studies also were carried out prior to the identification of additional variants with overlapping sequences. The present study using SYBR green qPCR overcomes these limitations and more precisely quantifies the expression of *OPRM1* splice variant mRNAs. Using these assays, we investigated the expression profiles of all *OPRM1* splice variant mRNAs in various brain regions of four inbred mouse strains, for the first time, a complete expression mRNA profile. However, even this approach does not address their expression at the protein level.

Although our current approach provides selectivity, dissecting tissues from a limited of brain regions does not provide anatomical resolution at the cellular level and provides only an incomplete assessment of variant expression. Future studies using single cell qPCR or more sophisticated *in situ* hybridization approaches may overcome these limitations. However, the current studies provide the first step toward our understanding of *OPRM1* splice variant expression in brain.

The abundance of the *OPRM1* splice variant mRNAs covers a wide range. These mRNA levels presumably reflect steady-state levels that depend upon many factors that may vary over time, including transcription, post-transcriptional modification and mRNA degradation. The *OPRM1* gene has two distinct promoters, exon 1 and exon 11 promoters. Most of the exon 1 promoter-controlled variants involve 3′ splicing, yielding unique C-terminal sequences, and are quite abundant. They include mMOR-1A, mMOR-1B1, mMOR-1C, mMOR-1*O* and mMOR-1V. In contrast, the exon 11 promoter-controlled variants generate 6-TM variants and are expressed at lower levels. While the promoters may influence mRNA expression, they cannot explain all the differences. Within each promoter group, mRNA levels vary, as shown by the far lower levels of mMOR-1B2 and mMOR-1P levels than those of mMOR-1A, mMOR-1B1 and mMOR-1C in the PAG, hypothalamus and hippocampus of B6 mice and the differences in the exon 11 promoter-controlled 6-TM variants mMOR-1G, mMOR-1K and mMOR-1N.

mRNA stability also may impact expression levels. Of particular interest is nonsense-mediated decay (NMD) that targets an mRNA containing a premature stop codon located more than 50 nt upstream of the last exon-exon junction [64], [65]. Eight variants, including mMOR-1E, mMOR-1F, mMOR-1Q, mMOR-1R, mMOR-1T, mMOR-1V, mMOR-1W, and mMOR-1Z, are potential targets of NMD. However, the variability of expression among these variants implies that NMD is not solely one factor among many influencing expression levels.

mRNA levels may not correlate with either their corresponding protein expression or with their pharmacological importance. For example, immunostaining by an exon 7/8/9-specific antisera that label mMOR-1C and mMOR-1M was as robust as that seen with the exon 4-specific antisera labeling mMOR-1, mMOR-1H, mMOR-1i and mMOR-1J despite great differences in their respective mRNA levels [23], [24]. The lack of correlation between mRNA and protein levels also may involve many factors. The association of the different mRNAs with polyribosomes can vary widely (Z. Lu, J. Xu, G.W. Pasternak, and Y.X. Pan, unpublished data), suggesting the differential regulation of the variants at the translational level. Other evidence indicates that the stability of the variants at the protein level is dependent upon a range of factors. For example, internalization and recycling of the receptors varies among the variants [31] and co-expression of single TM splice variants stabilizes MOR-1 protein levels, decreasing its degradation, extending its half-lives and thereby increasing its overall levels [48]. Therefore, it will be important to develop a reliable and an accurate method for determining protein expression of these OPRM1 splice variants in the future.

mRNA levels do not necessarily correlate with pharmacological relevance. For example, mMOR-1D has been implicated in morphine-induced itch through its dimerization with gastrin-releasing peptide receptor (GRPR) [66] despite its low mRNA levels. Exon 11-associated 6-TM variants are also among the low abundant transcripts. Yet, an exon 11 knockout mouse model indicates a key role of these 6-TM variants in the analgesic actions of a range of opioids, including heroin, morphine-6β-glucuronide, fentanyl, levorphanol and buprenorphine [33], [34]. These 6-TM variants also are critical in the actions of a novel class of opioid analgesic exemplified by 3′-iodobenzoyl-6β-naltexamide (IBNtxA), which is interesting since IBNtxA produces potent analgesia without many traditional opioid side-effects such as respiratory depression, physical dependence and reward behavior [34]. The importance of 6-TM variants is further supported by recent studies showing the rescue of IBNtxA analgesia in knockout mice lacking 6-TM variants that are insensitive to IBNtxA by the lentiviral-mediated delivery of a 6-TM variant, mMOR-1G (Z. Lu, J. Xu, G. Rossi, G.W. Pasternak, and Y.X. Pan, unpublished observations).

Our data also demonstrate variability in the expression of *OPRM1* splice variants in different strains. The importance of genetic backgrounds in opioid pharmacology is becoming increasingly appreciated. The four strains chosen for evaluation differ in their sensitivity to morphine analgesia, with AD_50_ values ranging from 6.8 mg/kg in C57BL/6 and 7.2 mg/kg in SJL to 13.7 mg/kg in 129P3 and 18.7 mg/kg in SWR mice [36]. They also differ in the development of tolerance to chronic morphine dosing of over three days, with analgesic shifts of 0.8 in the 129P3 mice to 2.2 in the SJL mice, 5 in SWR mice and 7.2 in the C56BL/6 mice [36]. Dependence, measured by jumping following naloxone precipitated withdrawal, also varies with the 129P3 and SJL mice showing virtually no jumping with moderate levels of jumping in the C57BL/6 mice and the most in the SWR mice [35]. Although four strains is a very limited sample, it is interesting that linear regression analysis correlating mRNA expression levels with 1) morphine analgesia using half-maximal analgesic doses (AD_50_), 2) morphine tolerance using the shift in AD_50_ fold with chronic dosing over three days or [36] or 3) physical dependence determined by jump scores from naloxone-precipitated withdrawal [35] revealed strong correlations for some of the variants (r^2^>0.90; Fig. S5), raising questions regarding the functional relevance of the variants in these brain regions. While intriguing, these correlations must be interpreted cautiously and require additional studies. Recently, we observed that long-term morphine treatment in CD-1 mice markedly upregulates a number of OPRM1 splice variants in various brain regions, as determined by the qPCR assays described here (J. Xu, A.J. Faskowitz, G.C. Rossi, M. Xu, Z. Lu, Y.-X Pan and G.W. Pasternak, submitted). Considering the variability in mRNA expression among the current strains of mice, it will be interesting to compare morphine effects on the expression of OPRM1 splice variants in these inbred mice.

Why the pattern of the variant expression in these four inbred mouse strains containing the same single-copy *OPRM1* gene varies is not clear. Presumably, the strains differ in their modulation of the promoters and alternative splicing machinery. These may reflect genetic variations, such as single nucleotide polymorphisms (SNPs), deletions, insertions and transposon elements. Recently, we identified a heroin addiction severity-associated intronic SNP that modulates alternative splicing of the human *OPRM1* variants via hnRNPH interaction [67]. Further identifying the genetic variations that modulate the strain-specific *OPRM1* alternative splicing will greatly advance our knowledge not only on the *OPRM1* alternative splicing, but also on alternative splicing in general.

## Supporting Information

Figure S1 **Expression levels of additional ten** ***OPRM1*** **splice variants in brain regions of B6, SJL and SWR mice.** Each panel represents the regional expressions of one variant in four inbred mouse strains. Red bar: B6 mice; Green bar: 129 mice; Blue bar: SJL mice; Brown bar: SWR mice. Bars represent the mean of E^−ΔC(t)^ values ± S.E.M. Pfc: prefrontal cortex; Str: striatum; Tha: thalamus; Hyp: hypothalamus; Hip: hippocampus; PAG: periaqueductal gray; BS: brainstem; Cb: cerebellum; Spc: spinal cord; WB: whole brain. Significant difference was calculated by Two-way ANOVA with Tukey’s multiple comparisons test for each variant. The results of the statistical analysis were listed in Table S3.
(TIF)

Figure S2 **Expression levels of additional nine** ***OPRM1*** **splice variants in brain regions of B6, SJL and SWR mice.** Each panel represents the regional expressions of one variant in four inbred mouse strains. Red bar: B6 mice; Green bar: 129 mice; Blue bar: SJL mice; Brown bar: SWR mice. Bars represent the mean of E^−ΔC(t)^ values ± S.E.M. Pfc: prefrontal cortex; Str: striatum; Tha: thalamus; Hyp: hypothalamus; Hip: hippocampus; PAG: periaqueductal gray; BS: brainstem; Cb: cerebellum; Spc: spinal cord; WB: whole brain. Significant difference was calculated by Two-way ANOVA with Tukey’s multiple comparisons test for each variant. The results of the statistical analysis were listed in Table S3.
(TIF)

Figure S3 **Strain-specific expressions of additional ten** ***OPRM1*** **splice variants.** Each panel represents the expression of one variant among four mouse strains in ten brain regions. Red bar: B6 mice; Green bar: 129 mice; Blue bar: SJL mice; Brown bar: SWR mice. Bars represent the mean of E^−ΔC(t)^ values ± S.E.M. Pfc: prefrontal cortex; Str: striatum; Tha: thalamus; Hyp: hypothalamus; Hip: hippocampus; PAG: periaqueductal gray; BS: brainstem; Cb: cerebellum; Spc: spinal cord; WB: whole brain. mE1-2 represents all full-length 7TM variants. Significant difference was calculated by Two-way ANOVA with Tukey’s multiple comparison for each variant. The results of the statistical analysis were listed in Table S3.
(TIF)

Figure S4 **Strain-specific expressions of additional nine** ***OPRM1*** **splice variants.** Each panel represents the expression of one variant among four mouse strains in ten brain regions. Red bar: B6 mice; Green bar: 129 mice; Blue bar: SJL mice; Brown bar: SWR mice. Bars represent the mean of E^−ΔC(t)^ values ± S.E.M. Pfc: prefrontal cortex; Str: striatum; Tha: thalamus; Hyp: hypothalamus; Hip: hippocampus; PAG: periaqueductal gray; BS: brainstem; Cb: cerebellum; Spc: spinal cord; WB: whole brain. Significant difference was calculated by Two-way ANOVA with Tukey’s multiple comparison for each variant. The results of the statistical analysis were listed in Table S3.
(TIF)

Figure S5 **Correlation of the expression levels of** ***OPRM1*** **splice variant mRNAs with morphine-induced analgesia, tolerance and physical dependence in four inbred mouse strains.** A & B: Correlation of the expression levels of *OPRM1* splice variant mRNAs with morphine analgesia in four inbred mouse strains. The square of the correlation coefficient (r^2^) listed was obtained from linear regression analysis (Prizm 5.0) between expression level (E^−ΔC(t)^ *10^4^) values (mean ± S.E.M.) for each variant in the indicated brain region and morphine analgesia potency (log (AD_50_)). The AD_50_ (mg/kg) values in B6, SJL, 129 and SWR mice were 6.8, 7.2, 13.7 and 18.7, respectively [36]. C & D: Correlation of the expression levels of *OPRM1* splice variant mRNAs with morphine tolerance in four inbred mouse strains. The square of the correlation coefficient (r^2^) listed was obtained from linear regression analysis (Prizm 5.0) between expression level (E^−ΔC(t)^ *10^4^) values (mean ± S.E.M.) for each variant in the indicated brain region and morphine AD_50_ shift before and after chronic morphine treatment (log (Morphine AD_50_ shift)). The AD_50_ shift values in 129, SJL, SWR and B6 mice were 0.8, 2.2, 5 and 7.2, respectively [36]. E & F: Correlation of the expression levels of *OPRM1* splice variant mRNAs with morphine-induced physical dependence in four inbred mouse strains. The square of the correlation coefficient (r^2^) listed was obtained from linear regression analysis (Prizm 5.0) between expression level (E^−ΔC(t)^ *10^4^) values (mean ± S.E.M.) for each variant in the indicated brain region and jumping scores after chronic morphine treatment followed by naloxone precipitated withdrawal (log (Jumps/15 min)). The jumping scores in 129, SJL, B6 and SWR mice were 7, 16, 60 and 200, respectively [35]. All the variants listed have an *r^2^* value over 0.90. A positive slope indicates that the more expression of the variants, the more AD_50_ values (the less potent) or the more AD_50_ value shifts (the more tolerant) or the more jumps (the more dependent) are. A negative slope shows opposite relationships. Pfc: prefrontal cortex; Str: striatum; Hyp: hypothalamus; Hip: hippocampus; PAG: periaqueductal gray; BS: brainstem; Spc: spinal cord; WB: whole brain.
(TIF)

Table S1 **SYBR green qPCR assay.** SYBR green qPCR assay for each variant was established as described in Materials and Methods. Optimized conditions including primers and their locations, thermal cycle condition, amplicon size and efficiency are listed. SP: sense primer; AP: antisense primer.
(XLSX)

Table S2 **Expression levels of OPRM1 splice variants.** Normalized expression level for each variant was calculated as E^−ΔC(t)^, where E is efficiency listed in Table S1, as described in Materials and Methods. The mean of E^−ΔC(t)^ values ± S.E.M. is listed.
(XLSX)

Table S3 **Two-Way ANOVA analysis.** Two-Way ANONVA analysis with Tukey’s multiple comparisons test was performed as described in Materials and Methods. Pfc: prefrontal cortex; Str: striatum; Tha: thalamus; Hyp: hypothalamus; Hip: hippocampus; PAG: periaqueductal gray; BS: brainstem; Cb: cerebellum; Spc: spinal cord; WB: whole brain. For each variant, left tables are regional comparisons in each inbred mouse strain, and right tables, strain comparisons in each brain region. *p* values listed in tables are: *: *p*<0.05; **: *p*<0.01; ***: *p*<0.001; ****: *p*<0.0001.
(XLSX)

## References

[1] Pasternak GW (1993) Pharmacological mechanisms of opioid analgesics. Clin Neuropharmacol 16: 1–18. 8093680 10.1097/00002826-199302000-00001

[2] Foley KM (1985) Management of Cancer Pain. In: DeVita VT, Hellman S, Rosenberg SA, editors. Cancer: Principles and Practice of Oncology. New York: Lippincott. 1940–1961.

[3] Payne R, Pasternak GW (1992) Pain. In: Johnston MV, Macdonald RL, Young AB, editors. Principles of drug therapy in neurology. Philadelphia: F.A. Davis. 268–301.

[4] Portenoy RK (1996) Opioid Analgesics. In: Portenoy RK, Kanner RM, editors. Pain management: Theory and practice. Philadelphia: F.A. Davis. 248–276.

[5] Ling GSF, MacLeod JM, Lee S, Lockhart SH, Pasternak GW (1984) Separation of morphine analgesia from physical dependence. Science 226: 462–464. 6541807 10.1126/science.6541807

[6] Ling GSF, Spiegel K, Lockhart SH, Pasternak GW (1985) Separation of opioid analgesia from respiratory depression: evidence for different receptor mechanisms. J Pharmacol Exp Ther 232: 149–155. 2981312

[7] Moulin DE, Ling GSF, Pasternak GW (1988) Unidirectional analgesic cross tolerance between morphine and levorphanol in the rat. Pain 33: 233–239. 2837716 10.1016/0304-3959(88)90095-4

[8] Chang A, Emmel DW, Rossi GC, Pasternak GW (1998) Methadone analgesia in morphine-insensitive CXBK mice. Eur J Pharmacol 351: 189–191. 9687002 10.1016/s0014-2999(98)00366-5

[9] Pasternak GW (2004) Multiple opiate receptors: deja vu all over again. Neuropharmacology 47 Suppl 1: 312–323. 15464147 10.1016/j.neuropharm.2004.07.004

[10] Leipzig J, Pevzner P, Heber S (2004) The Alternative Splicing Gallery (ASG): bridging the gap between genome and transcriptome. Nucleic Acids Res 32: 3977–3983. 15292448 10.1093/nar/gkh731 PMC506815

[11] Rossi GC, Pan YX, Brown GP, Pasternak GW (1995) Antisense Mapping the Mor-1 Opioid Receptor - Evidence for Alternative Splicing and A Novel Morphine-6-Beta-Glucuronide Receptor. FEBS Lett 369: 192–196. 7649256 10.1016/0014-5793(95)00757-z

[12] Rossi GC, Leventhal L, Pan YX, Cole J, Su W, et al (1997) Antisense mapping of MOR-1 in the rat: Distinguishing between morphine and morphine-6b-glucuronide antinociception. J Pharmacol Exp Ther 281: 109–114. 9103486

[13] Chen Y, Mestek A, Liu J, Hurley JA, Yu L (1993) Molecular cloning and functional expression of a m-opioid receptor from rat brain. Mol Pharmacol 44: 8–12. 8393525

[14] Wang JB, Imai Y, Eppler CM, Gregor P, Spivak CE, et al (1993) m opiate receptor: cDNA cloning and expression. Proc Natl Acad Sci USA 90: 10230–10234. 8234282 10.1073/pnas.90.21.10230 PMC47748

[15] Thompson RC, Mansour A, Akil H, Watson SJ (1993) Cloning and pharmacological characterization of a rat m opioid receptor. Neuron 11: 903–913. 8240812 10.1016/0896-6273(93)90120-g

[16] Pan YX (2005) Diversity and complexity of the mu opioid receptor gene: alternative pre-mRNA splicing and promoters. DNA Cell Biol 24: 736–750. 16274294 10.1089/dna.2005.24.736

[17] Pan Y-X, Pasternak GW (2010) Molecular Biology of Mu Opioid Receptors. In: Pasternak GW, editors. Opiate. Humana Press. 121–160.

[18] Kozak CA, Filie J, Adamson MC, Chen Y, Yu L (1994) Murine chromosomal location of the m and kappa opioid receptor genes. Genomics 21: 659–661. 7959748 10.1006/geno.1994.1331

[19] Giros B, Pohl M, Rochelle JM, Seldin MF (1995) Chromosomal localization of opioid peptide and receptor genes in the mouse. Life Sci 56: PL369–PL375. 7752808 10.1016/0024-3205(95)00119-q

[20] Belknap JK, Mogil JS, Helms ML, Richards SP, O'Toole LA, et al (1995) Localization to chromosome 10 of a locus influencing morphine analgesia in crosses derived from C57BL/6 and DBA/2 strains. Life Sci 57: L117–L124. 10.1016/0024-3205(95)02040-p 7643715

[21] Wang JB, Johnson PS, Persico AM, Hawkins AL, Griffin CA, et al (1994) Human m opiate receptor: cDNA and genomic clones, pharmacologic characterization and chromosomal assignment. FEBS Lett 338: 217–222. 7905839 10.1016/0014-5793(94)80368-4

[22] Pasternak GW, Pan YX (2013) Mu opioids and their receptors: evolution of a concept. Pharmacol Rev 65: 1257–1317 65/4/1257 [pii]; 10.1124/pr.112.007138 [doi] 24076545 PMC3799236

[23] Abbadie C, Pan YX, Pasternak GW (2000) Differential distribution in rat brain of mu opioid receptor carboxy terminal splice variants MOR-1C-like and MOR-1-like immunoreactivity: Evidence for region-specific processing. J Comp Neurol 419: 244–256. 10723002 10.1002/(sici)1096-9861(20000403)419:2<244::aid-cne8>3.0.co;2-r

[24] Abbadie C, Pan YX, Drake CT, Pasternak GW (2000) Comparative immunohistochemical distributions of carboxy terminus epitopes from the mu-opioid receptor splice variants MOR-1D, MOR-1 and MOR-1C in the mouse and rat CNS. Neuroscience 100: 141–153. 10996465 10.1016/s0306-4522(00)00248-7

[25] Pan YX, Xu J, Bolan EA, Abbadie C, Chang A, et al (1999) Identification and characterization of three new alternatively spliced mu opioid receptor isoforms. Mol Pharmacol 56: 396–403. 10419560 10.1124/mol.56.2.396

[26] Pan YX, Xu J, Bolan E, Chang A, Mahurter L, et al (2000) Isolation and expression of a novel alternatively spliced mu opioid receptor isoform, MOR-1F. FEBS Lett 466: 337–340. 10682855 10.1016/s0014-5793(00)01095-4

[27] Pan YX, Xu A, Mahurter L, Bolan E, Xu MM, et al (2001) Generation of the mu opioid receptor (MOR-1) protein by three new splice variants of the Oprm gene. Proc Natl Acad Sci USA 98: 14084–14089. 11717463 10.1073/pnas.241296098 PMC61171

[28] Pan L, Xu J, Yu R, Xu MM, Pan YX, et al (2005) Identification and characterization of six new alternatively spliced variants of the human mu opioid receptor gene, Oprm. Neuroscience 133: 209–220. 15893644 10.1016/j.neuroscience.2004.12.033

[29] Koch T, Schulz S, Schroder H, Wolf R, Raulf E, et al (1998) Carboxyl-terminal splicing of the rat mu opioid receptor modulates agonist-mediated internalization and receptor resensitization. J Biol Chem 273: 13652–13657. 9593704 10.1074/jbc.273.22.13652

[30] Abbadie C, Pasternak GW (2001) Differential in vivo internalization of MOR-1 and MOR-1C by morphine. Neuroreport 12: 3069–3072. 11568638 10.1097/00001756-200110080-00017

[31] Tanowitz M, Hislop JN, von ZM (2008) Alternative splicing determines the post-endocytic sorting fate of G-protein-coupled receptors. J Biol Chem 283: 35614–35621. 18936093 10.1074/jbc.M806588200 PMC2602910

[32] Bolan EA, Pasternak GW, Pan Y-X (2004) Functional analysis of MOR-1 splice variants of the mu opioid receptor gene, *Oprm* . Synapse 51: 11–18. 14579421 10.1002/syn.10277

[33] Pan YX, Xu J, Xu M, Rossi GC, Matulonis JE, et al (2009) Involvement of exon 11-associated variants of the mu opioid receptor MOR-1 in heroin, but not morphine, actions. Proc Natl Acad Sci U S A 106: 4917–4922 0811586106 [pii]; 10.1073/pnas.0811586106 [doi] 19273844 PMC2660730

[34] Majumdar S, Grinnell S, Le RV, Burgman M, Polikar L, et al (2011) Truncated G protein-coupled mu opioid receptor MOR-1 splice variants are targets for highly potent opioid analgesics lacking side effects. Proc Natl Acad Sci U S A 108: 19778–19783 1115231108 [pii]; 10.1073/pnas.1115231108 [doi] 22106286 PMC3241767

[35] Kest B, Palmese CA, Hopkins E, Adler M, Juni A, et al (2002) Naloxone-precipitated withdrawal jumping in 11 inbred mouse strains: evidence for common genetic mechanisms in acute and chronic morphine physical dependence. Neuroscience 115: 463–469. 12421612 10.1016/s0306-4522(02)00458-x

[36] Kest B, Hopkins E, Palmese CA, Adler M, Mogil JS (2002) Genetic variation in morphine analgesic tolerance: a survey of 11 inbred mouse strains. Pharmacol Biochem Behav 73: 821–828. 12213527 10.1016/s0091-3057(02)00908-5

[37] Klein G, Juni A, Waxman AR, Arout CA, Inturrisi CE, et al (2008) A survey of acute and chronic heroin dependence in ten inbred mouse strains: evidence of genetic correlation with morphine dependence. Pharmacol Biochem Behav 90: 447–452. 18472145 10.1016/j.pbb.2008.03.030 PMC3627368

[38] Metten P, Crabbe JC, Belknap JK (2009) Genetic correlates of morphine withdrawal in 14 inbred mouse strains. Drug Alcohol Depend 99: 123–131. 18774238 10.1016/j.drugalcdep.2008.07.006 PMC3573847

[39] Schlussman SD, Zhang Y, Hsu NM, Allen JM, Ho A, et al (2008) Heroin-induced locomotor activity and conditioned place preference in C57BL/6J and 129P3/J mice. Neurosci Lett 440: 284–288. 18579303 10.1016/j.neulet.2008.05.103 PMC2562215

[40] Belknap JK, Riggan J, Cross S, Young ER, Gallaher EJ, et al.. (1998) Genetic determinants of morphine activity and thermal responses in 15 inbred mouse strains. Pharmacol Biochem Behav 59: 353–360. S0091-3057(97)00421-8 [pii]. 10.1016/s0091-3057(97)00421-8 9476981

[41] Kolesnikov Y, Jain S, Wilson R, Pasternak GW (1998) Lack of morphine and enkephalin tolerance in 129/SvEv mice: evidence for a NMDA receptor defect. J Pharmacol Exp Ther 284: 455–459. 9454784

[42] Xu J, Xu M, Rossi GC, Kest B, Pasternak GW, Pan Y-X (2009) Differential expression of alternatively spliced variant mRNAs from the mu opioid receptor (OPRM1) gene in brain regions of four inbred mouse strains. Soc Neurosci 232.1/D14. 10.1371/journal.pone.0111267 PMC4208855 25343478

[43] Paxinos G, Franklin K (2000) Mouse brain in stereotaxic coordinates. 2d.

[44] Pfaffl MW (2001) A new mathematical model for relative quantification in real-time RT-PCR. Nucleic Acids Res 29: e45. 11328886 10.1093/nar/29.9.e45 PMC55695

[45] Vandesompele J, De PK, Pattyn F, Poppe B, Van RN, et al (2002) Accurate normalization of real-time quantitative RT-PCR data by geometric averaging of multiple internal control genes. Genome Biol 3: RESEARCH0034. 12184808 10.1186/gb-2002-3-7-research0034 PMC126239

[46] Pfaffl MW, Tichopad A, Prgomet C, Neuvians TP (2004) Determination of stable housekeeping genes, differentially regulated target genes and sample integrity: BestKeeper–Excel-based tool using pair-wise correlations. Biotechnol Lett 26: 509–515. 15127793 10.1023/b:bile.0000019559.84305.47

[47] Kaufman DL, Keith DE Jr, Anton B, Tian J, Magendzo K, et al (1995) Characterization of the murine m opioid receptor gene. J Biol Chem 270: 15877–15883. 7797593 10.1074/jbc.270.26.15877

[48] Xu J, Xu M, Brown T, Rossi GC, Hurd YL, et al (2013) Stabilization of the mu opioid receptor by truncated single transmembrane splice variants through a chaperone-like action. JBC 288: 21211–21227. 10.1074/jbc.M113.458687 PMC3774389 23760268

[49] Atweh SF, Kuhar MJ (1983) Distribution and physiological significance of opioid receptors in the brain. British Medical Bulletin 39: 47–52. 6301608 10.1093/oxfordjournals.bmb.a071789

[50] Atweh SF, Kuhar MJ (1977) Autoradiographic localization of opiate receptors in rat brain. III. The telencephalon. Brain Res 134: 393–405. 198065 10.1016/0006-8993(77)90817-4

[51] Atweh SF, Kuhar MJ (1977) Autoradiographic localization of opiate receptors in rat brain. I. Spinal cord and lower medulla. Brain Res 124: 53–67. 191149 10.1016/0006-8993(77)90863-0

[52] Atweh SF, Kuhar MJ (1977) Autoradiographic localization of opiate receptors in rat brain. II. The brain stem. Brain Res 129: 1–12. 194658 10.1016/0006-8993(77)90965-9

[53] Mansour A, Khachaturian H, Lewis ME, Akil H, Watson SJ (1987) Autoradiographic differentiation of mu, delta, and kappa opioid receptors in the rat forebrain and midbrain. J Neurosci 7: 2445–2464. 3039080 PMC6568954

[54] Mansour A, Fox CA, Burke S, Akil H, Watson SJ (1995) Immunohistochemical localization of the cloned m opioid receptor in the rat CNS. J Chem Neuroanat 8: 283–305. 7669273 10.1016/0891-0618(95)00055-c

[55] Mansour A, Fox CA, Burke S, Watson SJ (1994) Immunohistochemical localization of the mu opioid receptors. Regul Pept 54: 179–180.

[56] Mansour A, Fox CA, Burke S, Meng F, Thompson RC, et al (1994) Mu, delta, and kappa opioid receptor mRNA expression in the rat CNS: An in situ hybridization study. J Comp Neurol 350: 412–438. 7884049 10.1002/cne.903500307

[57] Mansour A, Fox CA, Akil H, Watson SJ (1995) Opioid-receptor mRNA expression in the rat CNS: Anatomical and functional implications. Trends Neurosci 18: 22–29. 7535487 10.1016/0166-2236(95)93946-u

[58] Mansour A, Fox CA, Thompson RC, Akil H, Watson SJ (1994) m-Opioid receptor mRNA expression in the rat CNS: comparison to m-receptor binding. Brain Res 643: 245–265. 8032920 10.1016/0006-8993(94)90031-0

[59] Arvidsson U, Riedl M, Chakrabarti S, Lee J-H, Nakano AH, et al (1995) Distribution and targeting of a m-opioid receptor (MOR1) in brain and spinal cord. J Neurosci 15: 3328–3341. 7751913 10.1523/JNEUROSCI.15-05-03328.1995 PMC6578209

[60] Honda CN, Arvidsson U (1995) Immunohistochemical localization of delta- and mu-opioid receptors in primate spinal cord. Neuroreport 6: 1025–1028. 7632887 10.1097/00001756-199505090-00019

[61] Ding YQ, Kaneko T, Nomura S, Mizuno N (1996) Immunohistochemical localization of m-opioid receptors in the central nervous system of the rat. J Comp Neurol 367: 375–402. 8698899 10.1002/(SICI)1096-9861(19960408)367:3<375::AID-CNE5>3.0.CO;2-2

[62] Moriwaki A, Wang JB, Svingos A, Van Bockstaele E, Cheng P, et al (1996) m Opiate receptor immunoreactivity in rat central nervous system. Neurochem Res 21: 1315–1331. 8947922 10.1007/BF02532373

[63] Wang H, Moriwaki A, Wang JB, Uhl GR, Pickel VM (1997) Ultrastructural immunocytochemical localization of mu-opioid receptors in dendritic targets of dopaminergic terminals in the rat caudate- putamen nucleus. Neuroscience 81: 757–771. 9316027 10.1016/s0306-4522(97)00253-4

[64] Lejeune F, Maquat LE (2005) Mechanistic links between nonsense-mediated mRNA decay and pre-mRNA splicing in mammalian cells. Current Opinion in Cell Biology 17: 309–315. 15901502 10.1016/j.ceb.2005.03.002

[65] Chang YF, Imam JS, Wilkinson MF (2007) The nonsense-mediated decay RNA surveillance pathway. Annu Rev Biochem 76: 51–74 10.1146/annurev.biochem.76.050106.093909 [doi] 17352659

[66] Liu XY, Liu ZC, Sun YG, Ross M, Kim S, et al (2011) Unidirectional cross-activation of GRPR by MOR1D uncouples itch and analgesia induced by opioids. Cell 147: 447–458 S0092-8674(11)01064-6 [pii]; 10.1016/j.cell.2011.08.043 [doi] 22000021 PMC3197217

[67] Xu J, Lu Z, Xu M, Pan L, Deng Y, et al (2014) A Heroin Addiction Severity-Associated Intronic Single Nucleotide Polymorphism Modulates Alternative Pre-mRNA Splicing of the mu Opioid Receptor Gene OPRM1 via hnRNPH Interactions. J Neurosci 34: 11048–11066 34/33/11048 [pii]; 10.1523/JNEUROSCI.3986-13.2014 [doi] 25122903 PMC4131016

