# Supplementary figures and images for: Differential Expressions of the Alternatively Spliced Variant mRNAs of the µ Opioid Receptor Gene, OPRM1, in Brain Regions of Four Inbred Mouse Strains
Source: PLoS One. 2014 Oct 24;9(10):e111267. doi: 10.1371/journal.pone.0111267 (PMC4208855; doi:10.1371/journal.pone.0111267)

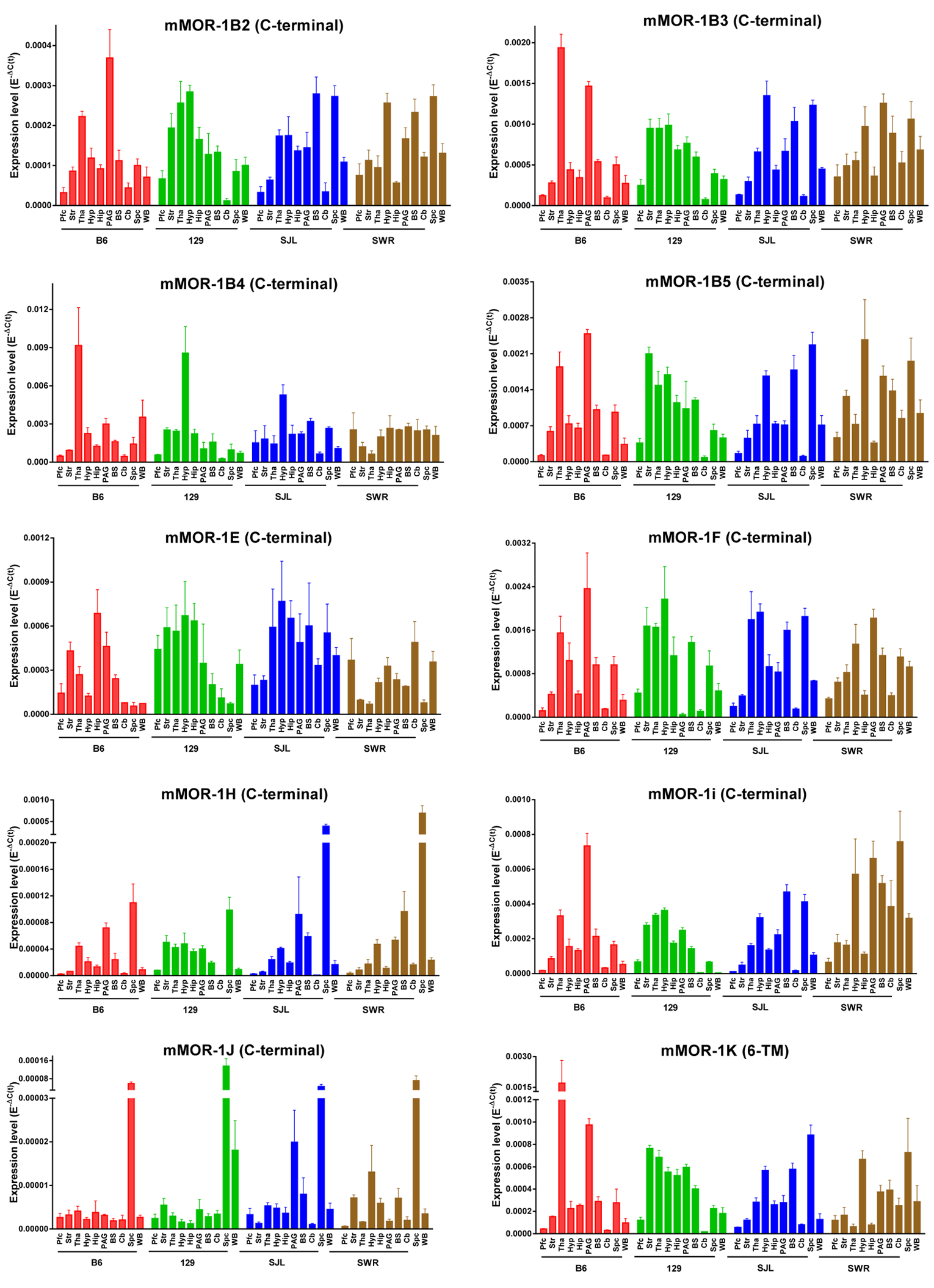

Supplement: Figure S1 — Expression levels of additional ten OPRM1 splice variants in brain regions of B6, SJL and SWR mice. Each panel represents the regional expressions of one variant in four inbred mouse strains. Red bar: B6 mice; Green bar: 129 mice; Blue bar: SJL mice; Brown bar: SWR mice. Bars represent the mean of E−ΔC(t) values ± S.E.M. Pfc: prefrontal cortex; Str: striatum; Tha: thalamus; Hyp: hypothalamus; Hip: hippocampus; PAG: periaqueductal gray; BS: brainstem; Cb: cerebellum; Spc: spinal cord; WB: whole brain. Significant difference was calculated by Two-way ANOVA with Tukey’s multiple comparisons test for each variant. The results of the statistical analysis were listed in Table S3. (TIF) [file pone.0111267.s001.tif]

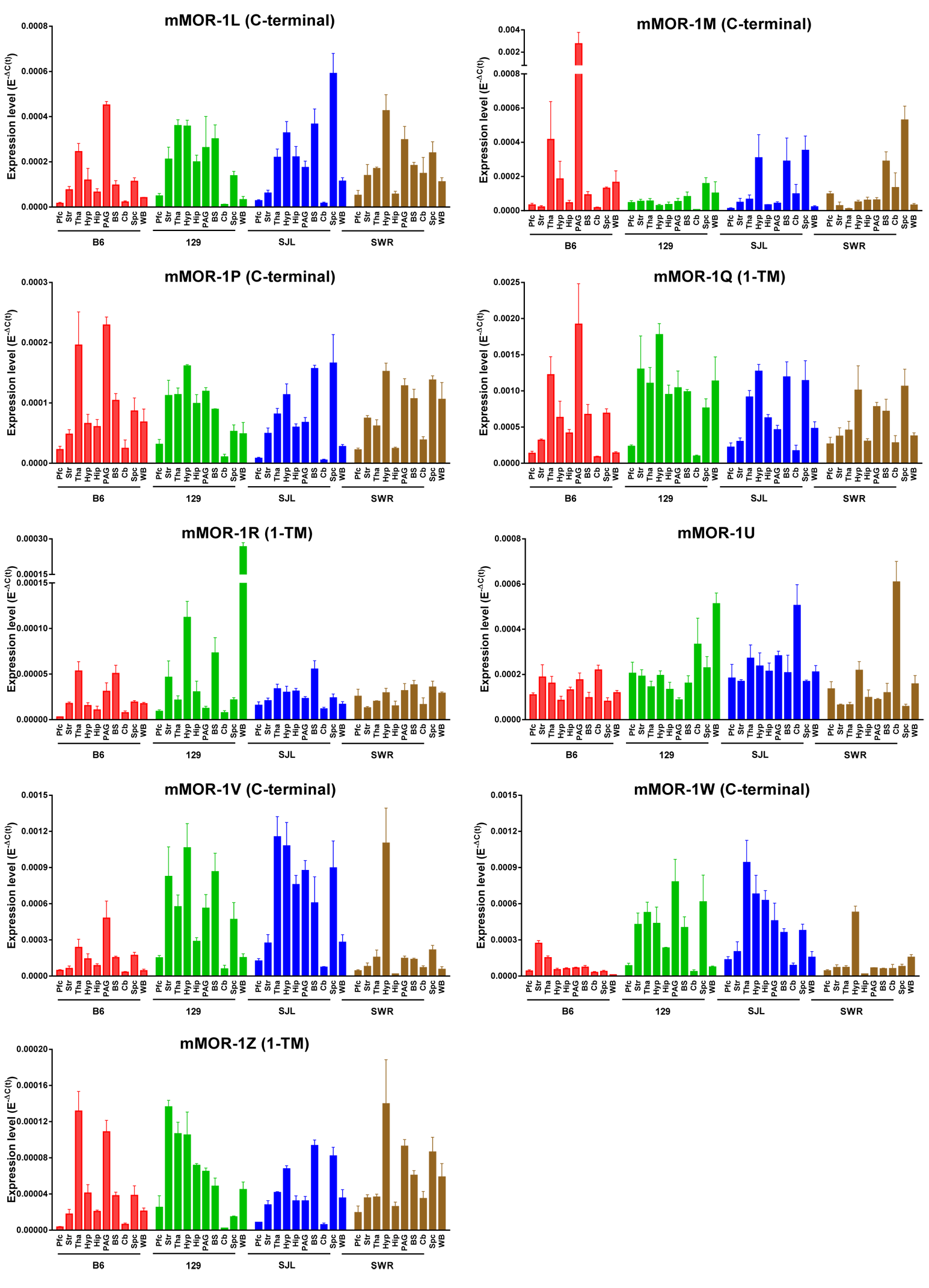

Supplement: Figure S2 — Expression levels of additional nine OPRM1 splice variants in brain regions of B6, SJL and SWR mice. Each panel represents the regional expressions of one variant in four inbred mouse strains. Red bar: B6 mice; Green bar: 129 mice; Blue bar: SJL mice; Brown bar: SWR mice. Bars represent the mean of E−ΔC(t) values ± S.E.M. Pfc: prefrontal cortex; Str: striatum; Tha: thalamus; Hyp: hypothalamus; Hip: hippocampus; PAG: periaqueductal gray; BS: brainstem; Cb: cerebellum; Spc: spinal cord; WB: whole brain. Significant difference was calculated by Two-way ANOVA with Tukey’s multiple comparisons test for each variant. The results of the statistical analysis were listed in Table S3. (TIF) [file pone.0111267.s002.tif]

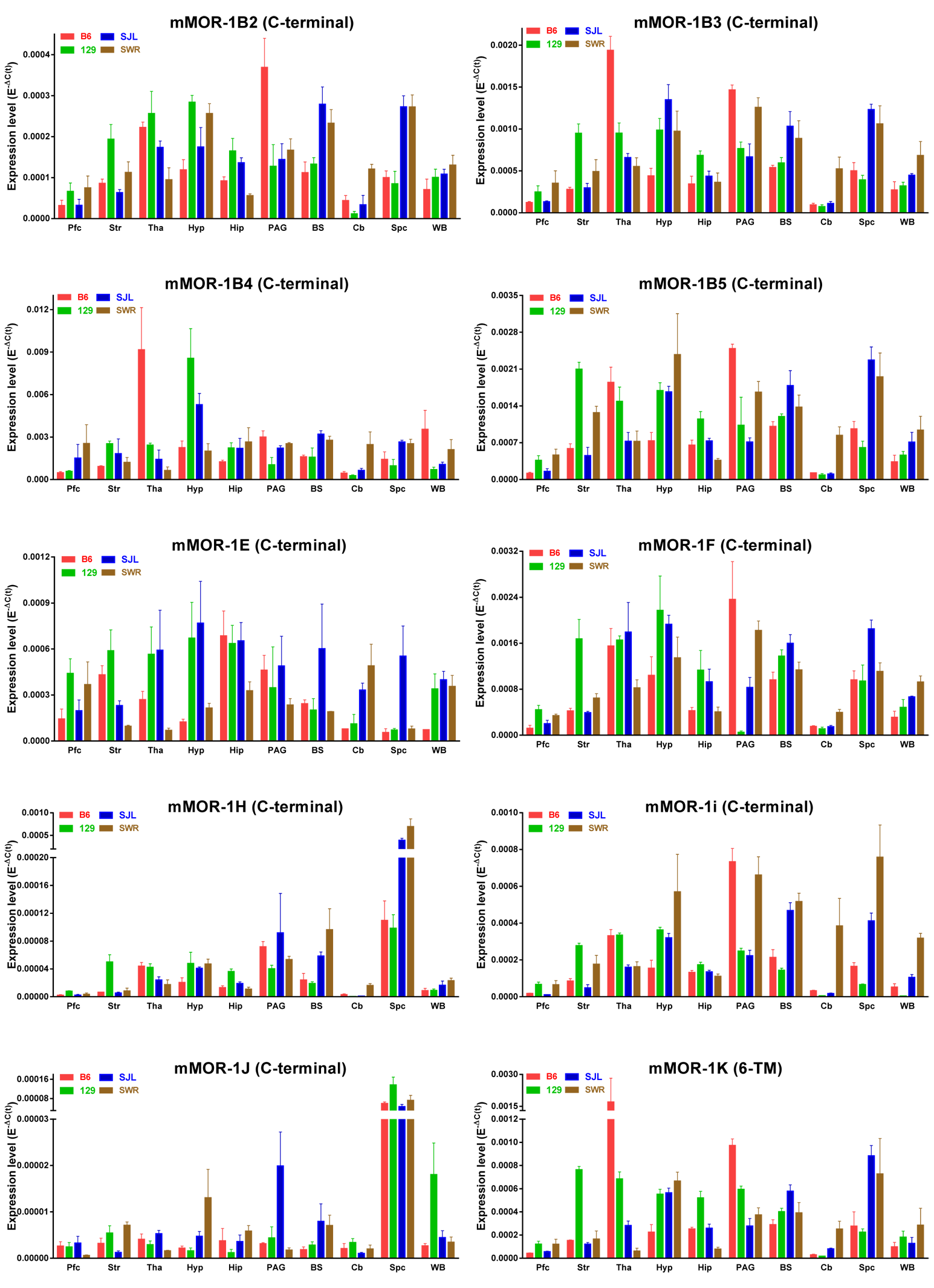

Supplement: Figure S3 — Strain-specific expressions of additional ten OPRM1 splice variants. Each panel represents the expression of one variant among four mouse strains in ten brain regions. Red bar: B6 mice; Green bar: 129 mice; Blue bar: SJL mice; Brown bar: SWR mice. Bars represent the mean of E−ΔC(t) values ± S.E.M. Pfc: prefrontal cortex; Str: striatum; Tha: thalamus; Hyp: hypothalamus; Hip: hippocampus; PAG: periaqueductal gray; BS: brainstem; Cb: cerebellum; Spc: spinal cord; WB: whole brain. mE1-2 represents all full-length 7TM variants. Significant difference was calculated by Two-way ANOVA with Tukey’s multiple comparison for each variant. The results of the statistical analysis were listed in Table S3. (TIF) [file pone.0111267.s003.tif]

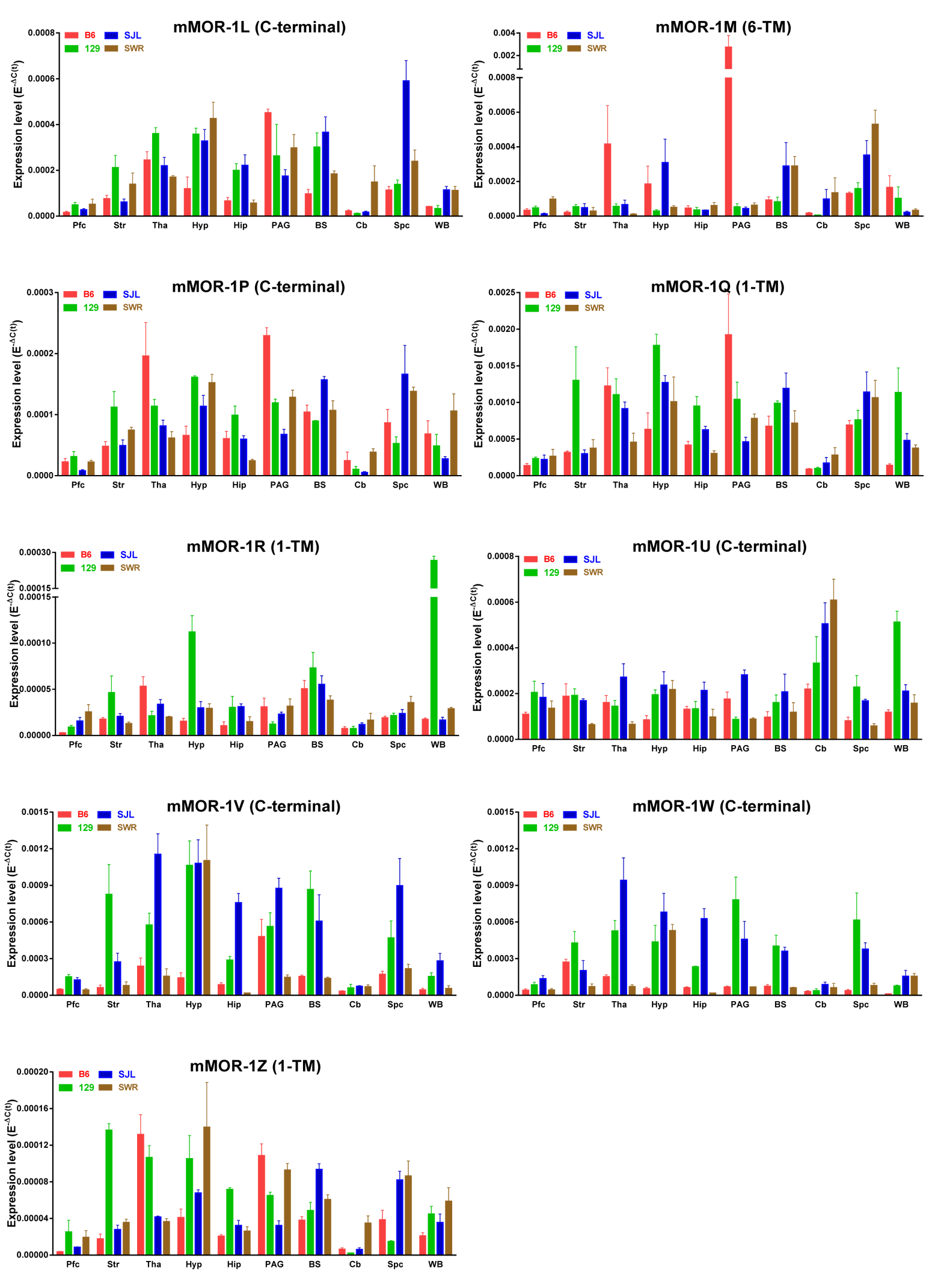

Supplement: Figure S4 — Strain-specific expressions of additional nine OPRM1 splice variants. Each panel represents the expression of one variant among four mouse strains in ten brain regions. Red bar: B6 mice; Green bar: 129 mice; Blue bar: SJL mice; Brown bar: SWR mice. Bars represent the mean of E−ΔC(t) values ± S.E.M. Pfc: prefrontal cortex; Str: striatum; Tha: thalamus; Hyp: hypothalamus; Hip: hippocampus; PAG: periaqueductal gray; BS: brainstem; Cb: cerebellum; Spc: spinal cord; WB: whole brain. Significant difference was calculated by Two-way ANOVA with Tukey’s multiple comparison for each variant. The results of the statistical analysis were listed in Table S3. (TIF) [file pone.0111267.s004.tif]

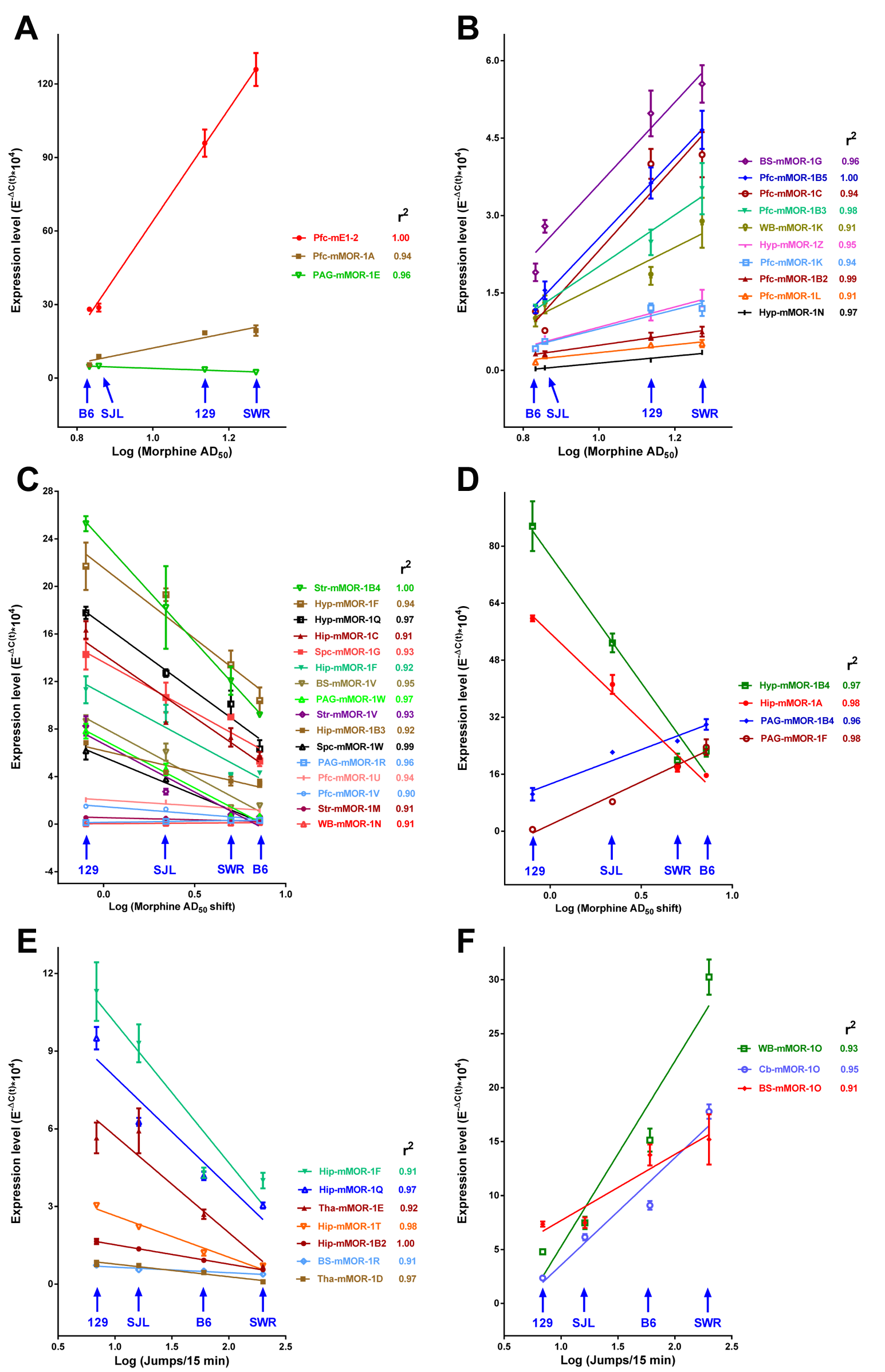

Supplement: Figure S5 — Correlation of the expression levels of OPRM1 splice variant mRNAs with morphine-induced analgesia, tolerance and physical dependence in four inbred mouse strains. A & B: Correlation of the expression levels of OPRM1 splice variant mRNAs with morphine analgesia in four inbred mouse strains. The square of the correlation coefficient (r2) listed was obtained from linear regression analysis (Prizm 5.0) between expression level (E−ΔC(t) *104) values (mean ± S.E.M.) for each variant in the indicated brain region and morphine analgesia potency (log (AD50)). The AD50 (mg/kg) values in B6, SJL, 129 and SWR mice were 6.8, 7.2, 13.7 and 18.7, respectively [36]. C & D: Correlation of the expression levels of OPRM1 splice variant mRNAs with morphine tolerance in four inbred mouse strains. The square of the correlation coefficient (r2) listed was obtained from linear regression analysis (Prizm 5.0) between expression level (E−ΔC(t) *104) values (mean ± S.E.M.) for each variant in the indicated brain region and morphine AD50 shift before and after chronic morphine treatment (log (Morphine AD50 shift)). The AD50 shift values in 129, SJL, SWR and B6 mice were 0.8, 2.2, 5 and 7.2, respectively [36]. E & F: Correlation of the expression levels of OPRM1 splice variant mRNAs with morphine-induced physical dependence in four inbred mouse strains. The square of the correlation coefficient (r2) listed was obtained from linear regression analysis (Prizm 5.0) between expression level (E−ΔC(t) *104) values (mean ± S.E.M.) for each variant in the indicated brain region and jumping scores after chronic morphine treatment followed by naloxone precipitated withdrawal (log (Jumps/15 min)). The jumping scores in 129, SJL, B6 and SWR mice were 7, 16, 60 and 200, respectively [35]. All the variants listed have an r2 value over 0.90. A positive slope indicates that the more expression of the variants, the more AD50 values (the less potent) or the more AD50 value shifts (the more tolerant) or the more [file pone.0111267.s005.tif]
